# Prescribing patterns in people living with dementia in the community: A cross‐sectional study

**DOI:** 10.1111/ajag.13380

**Published:** 2024-10-27

**Authors:** Edward Chun Yin Lau, Yun‐Hee Jeon, Sarah N. Hilmer, Edwin C. K. Tan

**Affiliations:** ^1^ Faculty of Medicine and Health, School of Pharmacy The University of Sydney Sydney New South Wales Australia; ^2^ Faculty of Medicine and Health, Susan Wakil School of Nursing and Midwifery The University of Sydney Sydney New South Wales Australia; ^3^ Kolling Institute The University of Sydney and Northern Sydney Local Health District St Leonards New South Wales Australia

**Keywords:** aged, dementia, physician prescribing patterns, polypharmacy

## Abstract

**Objectives:**

To identify the prevalence of and factors associated with medication use in people living with dementia in the community.

**Methods:**

A cross‐sectional study using baseline data from a randomised controlled trial known as the Interdisciplinary Home‐bAsed Reablement Program (I‐HARP) between 2018 and 2021 in Sydney, Australia. Participants included people with mild–moderate dementia and their carers. Medication use was classified according to the Anatomical Therapeutic Chemical codes, while potentially inappropriate medications (PIMs) were defined using 2019 Beer's Criteria and 2024 Australian list. Logistic regression models were used to identify factors associated with use of medication classes.

**Results:**

A total of 130 people with dementia and their carers were included. Of the people with dementia, 35% were using antidementia medication, 48% psychotropics, 76% PIMs and 65% polypharmacy (≥5 medications). Polypharmacy was associated with the use of psychotropics (adjusted OR [aOR]: 5.09, 95% confidence interval [CI]: 1.94–13.39) and PIMs (aOR: 17.38, 95% CI: 5.12–59.02). Higher education level was associated with lower odds of psychotropic use (aOR: .33, 95% CI: .15–.76), and age over 80 years was associated with lower odds of antidementia medication use (aOR: .29; 95% CI: .12–.72).

**Conclusions:**

The use of PIMs, psychotropics and polypharmacy were common in this sample of people with dementia living in the community. Associations were seen between participant characteristics and medication use. Future research should focus on reviewing PIMs and polypharmacy in people with dementia living in the community to assess the impact on health outcomes.

**Policy Impact**

This study found polypharmacy and potentially inappropriate medications (PIMs) are common in Australians living with dementia in the community. Further research into developing and implementing strategies to reduce the burden of polypharmacy and PIMs in this vulnerable population is needed.

## 1 INTRODUCTION

Dementia is a neurodegenerative disorder that affects an estimated 472,000 people in Australia, and this is projected to more than double by 2058.^1^ People with dementia are high users of medications, for management of both dementia symptoms and comorbidities, due to the high rate of multimorbidity in this population. Currently, antidementia medications, such as cholinesterase inhibitors and memantine, may slow down functional and cognitive decline in people with dementia but do not modify the pathology of dementia.^2^ Previous research has shown that over half of people with dementia are exposed to polypharmacy and potentially inappropriate medications (PIMs),^3^, ^4^ which are associated with adverse health outcomes.^5^ Furthermore, people with dementia have a reduced ability to manage their medication regimens due to cognitive and functional impairment.^6^ People living with dementia in the community are at a high risk of poisoning and adverse drug events,^7^, ^8^ which can lead to hospitalisation, residential aged care home admission or mortality. This highlights the importance of examining prescribing patterns to ensure the quality and judicious use of medications in this population.^8^

Previous research in Australia has investigated prescribing patterns of people with dementia living in residential aged care facilities (RACFs) or focused on particular drug classes.^9^, ^10^ While previous international studies have found high rates of psychotropic use in people with dementia living in the community,^11^, ^12^ such research is limited in Australia. With treatment guidelines and sociodemographic factors often varying between different countries, it is important to understand the local prescribing patterns in people with dementia. Therefore, this study aimed to investigate the prevalence and factors associated with medication use in people living with dementia in the Australian community.

## 2 METHODS

### 2.1 Data source, study design and population

Data for this study were sourced from the Interdisciplinary Home‐bAsed Reablement Program (I‐HARP) trial, which is a 12‐month multicentre pragmatic randomised controlled trial to evaluate the effectiveness of a home‐based rehabilitation program designed to improve the functional independence of people with mild–moderate dementia. The I‐HARP consists of up to 12 home‐visits by a registered nurse and an occupational therapist, and other allied health if needed; home modifications; and carer support.^13^ Data were collected between 2018 and 2021. Participants were dyads of people aged over 60 with mild‐to‐moderate dementia, and their primary carers. Clinical assessors assessed the capacity of people with dementia and their carers to consent, with written consent being obtained from both client and carer individually thereafter. Cholinesterase inhibitor users were excluded if their doses have not been stable for at least 3 months. Participants were recruited with their carers from a pool of clients eligible for Federal Government‐supported community aged home care programs from two participating aged care providers and four participating hospital geriatric services in Sydney, Australia. Further inclusion and exclusion criteria for I‐HARP are available in the study protocol.^13^ All participants recruited into the I‐HARP program (130 dyads) were included in this cross‐sectional study. Baseline characteristics and medication data were collected by clinical assessors on the day of baseline assessment. The I‐HARP trial has been registered with the Australian New Zealand Clinical Trial Registry (ANZCTR: 12618000600246) and approved by the Sydney Local Health District Human Research Ethics Committee—Concord Repatriation General Hospital (HREC/18/CRGH/11).

### 2.2 Participant characteristics

#### 2.2.1 Demographic information

Demographic information, such as age, comorbidities, sex and level of education, were collected from people with dementia and their carers at baseline. Education level was defined as highest education level attained, categorised as less than high school certificate (HSC) or HSC and above. Selected comorbidities were captured (Table 1).

**TABLE 1:** Characteristics of study participants with dementia and their carers.

Participant characteristics (*n* = 130)	
Female, *n* (%)	76 (59)
Age, mean (SD)	81.4 (6.6)
Education level^a^, *n* (%)	
HSC or higher	66 (52)
Speaking English at home^b^, *n* (%)	119 (92)
Marital status^a^, *n* (%)	
Married or with partner	81 (63)
Pensioner concession card holder, *n* (%)	65 (50)
Medical history	
Head injury with loss of consciousness	11 (9)
Stroke	19 (15)
Meningitis, encephalitis, brain infections	1 (1)
Epilepsy, seizures, and convulsions	3 (2)
Heart attack	18 (14)
Parkinson's disease	3 (2)
Brain nerve trauma	19 (15)
Cancer	22 (17)
Mental health	28 (22)
Other	78 (60)
At least one comorbidity	113 (87)
Type of dementia, *n* (%)	
Alzheimer's disease	44 (34)
Vascular dementia	17 (13)
Frontotemporal dementia	9 (7)
Lewy body disease	3 (2)
Mixed	6 (5)
Other	51 (39)
Degree of dementia severity, *n* (%)	
Mild (GDRS 4)	73 (56)
Moderate (GDRS 5)	45 (35)
Moderate to moderately severe (GDRS5/6)	9 (7)
Moderately severe (GDRS 6)	3 (2)
Geriatric depression scale^a^, *n* (%)	
No depression	61 (48)
Suggestive depression	44 (34)
Depression	23 (18)
Functional ability (DAD), mean (SD)	59.79 (23.35)
Physical function (SPPB), median (IQR)	7 (5–9)
Weighted EQ‐5D‐5L, median (IQR)	.83 (.58–1.00)
Number of medications, mean (SD; range)	6.2 (3.7; 0–18)
Polypharmacy, *n* (%)	85 (65)
At least 1 PIM, *n* (%)	99 (76)
Carer characteristics (*n* = 130)	
Female, *n* (%)	91 (71)
Age, mean (SD)	66.8 (13.4)
Education level, *n* (%)	
HSC or higher	99 (76)
Zarit burden index^a^, mean (SD)	31.0 (13.4)
Weighted EQ‐5D‐5L^b^, median (IQR)	.75 (.59–.92)

Abbreviations: DAD, Disability assessment for dementia; GDRS, The global deterioration scale; HSC, Higher School Certificate; IQR, Inter‐quartile range; PIM, potentially inappropriate medication; SD, standard deviation; SPPB, short physical performance battery.

^a^ Two missing values.

^b^ Three missing values.

#### 2.2.2 Functional independence

Disability Assessment for Dementia (DAD) was used to examine the functional ability of people living with dementia.^14^ The person's ability to perform basic and instrumental activities of daily living and leisure activities was assessed by clinicians based on the carer interview.^14^ The score of the assessment ranged from 0 to 100 with a higher score on DAD equating to better functional ability.^14^

#### 2.2.3 Dementia severity

The Global Deterioration Scale (GDRS) was used to assess the level of dementia severity, based on the carer interview regarding the person's clinical characteristics of dementia, with a higher score indicating poorer cognitive function.^15^ Levels 4, 5, 6 and 7 indicate mild, moderate, moderately severe and severe dementia, respectively.^15^

#### 2.2.4 Physical function

The Short Physical Performance Battery (SPPB) was administered by clinicians to measure the physical function of people with dementia.^16^ SPPB involves a group of measurements, including gait speed, chair stand and balance tests^16^; a higher SPPB score is indicative of better physical function. Median split was used to categorise higher and lower physical function in this cohort.

#### 2.2.5 Depressive symptoms

The Collateral Source version of the Geriatric Depression Scale (CS‐GDS‐15) is a 15‐item instrument to measure the likelihood of depression in older adults; the score ranges from 0 to 15, with a higher score indicating a higher likelihood of a major depressive disorder and increased symptomatology.^17^ CS‐GDS‐15 was used to evaluate depressive symptoms in people with dementia based on carer interview; scores of over five and 10 are suggestive and highly suggestive of depression, respectively.^17^

#### 2.2.6 Health‐related quality of life (HrQoL)

EuroQol five dimensions (EQ‐5D‐5L) is a widely used instrument to measure HrQoL, which involves five levels of severity in five different dimensions (including mobility, self‐care, usual activities, pain/discomfort and anxiety/depression), and was included as a self‐administered measure of HrQoL. The EQ‐5D‐5L scores were weighted using an algorithm created by Norman et al., a lower score indicating poorer HrQoL.^18^

#### 2.2.7 Carer burden

The Zarit Burden Index (ZBI) is widely used to measure the level of carer burden with a 22‐item questionnaire.^19^ The score of this self‐administered test ranges from 0 to 88 with a higher score indicating greater burden experienced by the carer.^19^

### 2.3 Medication outcomes

Baseline prescription, over‐the‐counter and complementary or alternative medication usage by participants was collected and verified by trial assessors through multiple sources such as interview with people with dementia and carers, and observation of medication packages and lists in the baseline assessment. All medications used were recorded, and data including active ingredients, dosage and frequencies were collected. All medication use, including any dosage form, regular and pro re nata, was included in this study. The study protocol for data collection required trained trial assessors to prioritise medication data collections for psychotropics and other prescription medications over complementary medications and over‐the‐counter medications. Medications were coded according to the Anatomical Therapeutic Chemical (ATC) classification system. The total number of medications for each client was calculated by number of products. Any medications used were included in the count for polypharmacy, which was defined as five or more medications used concurrently during the baseline assessment. Psychotropic polypharmacy was defined as using two or more psychotropic medications. Potentially inappropriate medications in people with dementia were defined using two criteria: the 2019 Beer's criteria and 2024 Australian PIM list.^20^, ^21^ Both criteria contain a list of medication deemed potentially inappropriate if prescribed to older adults, and an additional list for people living with dementia.^20^, ^21^ Beer's criteria were further supplemented using an extensive list of anticholinergic medications from a previous literature review.^22^

The use of antidementia medications, psychotropics and PIMs in people with dementia were included in the analysis for the association between patient characteristics and medication usage. Detailed ATC codes are reported in Table S1. Common medications and combinations are reported descriptively.

### 2.4 Statistical analyses

Characteristics of participants (people living with dementia and their carers) and the prevalence of commonly used medications in people living with dementia were reported descriptively. Logistic regression was used to examine factors associated with the use of antidementia medications, psychotropics and PIMs. Explanatory variables that may impact medication usage in people with dementia based on clinical reasoning and previous literature were examined using binary logistic regression; these included characteristics of people with dementia including, age, sex, education, dementia severity (using GDRS), polypharmacy, depressive symptoms (using CS‐GDS‐15), physical function (using SPPB), functional ability (using DAD) and HrQoL (using EQ‐5D‐5L); and carer characteristics including carer burden (using ZBI) and carer HrQoL (using EQ‐5D‐5L).^11^, ^12^ Of the variables included in the logistic regression models, functional ability, HrQoL and carer burden were included as continuous variables while the others were included as binary categorical variables. Comorbidities were excluded from logistic regression models due to concerns of incomplete information and multicollinearity as high intercorrelations were found between polypharmacy and co‐morbidity. Missing values were handled using listwise deletion. Findings were deemed statistically significant when *p* < .05; no adjustment for multiple comparisons was made. Regression models were tested to ensure the assumption of a logistic regression model was met. All statistical analyses were performed using the IBM SPSS statistics for Windows, version 28.0 (IBM Corp., Armonk, NY, USA).

## 3 RESULTS

### 3.1 Characteristics of dyads

Baseline characteristics of client–carer dyads are summarised in Table 1. Of the participants with dementia, 59% were female and the mean age was 81.4 (standard deviation [SD] = 6.6) years. Over half of participants with dementia had mild dementia (*n* = 73, 56%). Of the carers, 71% were female, and the mean age was 66.8 (SD = 13.4) years. The mean ZBI score for carers was 31.0 (SD = 13.4).

### 3.2 Prescribing patterns in people with dementia

Prescribing patterns in people with dementia are shown in Table 2. Overall, 85 (65%) people with dementia were exposed to polypharmacy. Cardiovascular medications were the most widely used (*n* = 103, 79%), followed by nervous system medications (*n* = 94, 72%), medications for alimentary tract and metabolism (*n* = 82, 63%) and medications for blood and blood forming organs (*n* = 65, 50%). Medication use in people with dementia by ATC level 1 is shown in Table S2. Antidementia medication and psychotropics were used by 35% and 48% of people with dementia, respectively, and 16% of people with dementia were taking antidementia medication and psychotropics concurrently. Cholinesterase inhibitors were the most common antidementia treatment, of which donepezil was used most frequently (25%), while only six people with dementia used memantine, of whom four used a cholinesterase inhibitor concurrently. Concurrent use of cholinesterase inhibitors and anticholinergics was found in 28 (22%) participants. The prescribing pattern of antidementia medications in different types of dementia is shown in Table 3. Majority of participants using memantine were also using a cholinesterase inhibitor. The 20 most frequently used medications by people with dementia are shown in Table S3. Antidepressants were the most commonly used psychotropics (34%), followed by antipsychotics (9%); 12% of people with dementia were exposed to psychotropic polypharmacy characterised by the concurrent use of at least two psychotropics. A quarter of people with dementia were using at least one proton pump inhibitor (PPI) (29%) while 12% and 9% of people with dementia used PPIs concurrently with antidementia medications and NSAIDs, respectively. Overall, 73% and 35% of people with dementia used a PIM defined by Beer's Criteria and the Australian list, respectively; 76% of them were using at least one PIM defined by either list.

**TABLE 2:** Prescribing patterns of commonly used medication classes and combinations.

Drugs	*n* = 130	%
Any alimentary tract and metabolism (ATC A)	82	63
Any drugs for acid related disorders (ATC A02)	38	29
Any proton pump inhibitors (A02BC)	37	29
Any drugs for diabetes (A10)	21	16
Any vitamins and minerals supplements (A11, A12)	50	39
Any blood and blood forming organs (ATC B)	65	50
Any antiplatelet (B01AC)	47	36
Any anticoagulant (B01AA, B01AB, B01AE, B01AF)	16	12
Any anti anaemic preparations (B03)	7	5
Any cardiovascular drugs (ATC C)	103	79
Any antihypertensives (ATC C02)	7	5
Any diuretics (ATC C03)	16	12
Any beta‐blocking agents (ATC C07)	21	16
Any calcium channel blockers (ATC C08)	22	17
Any agents acting on the renin‐angiotensin system (ATC C09)	54	42
Any lipid‐modifying agent (ATC C10)	74	57
Any antidementia medications (N06D)	45	35
Any cholinesterase inhibitor (N06DA)	43	33
Donepezil (N06DA02)	33	25
Galantamine (N06DA04)	6	5
Rivastigmine (N06DA03)	4	3
Memantine (N06DX03)	6	5
Any cholinesterase inhibitor + memantine	4	3
Any psychotropics (N06A, N05A, N03A, N05B, N05C, N02A)	62	48
Any antidepressants (N06A)	48	34
SSRI (N06AB)	24	19
SNRI^a^	7	5
TCA^b^	4	3
Other antidepressants^c^	13	10
Any antipsychotics (N05A)	11	9
Any anxiolytics (N05B)	3	2
Any antiepileptics (N03A)	8	6
Any opioids (N02A)	6	5
Any benzodiazepines and Z‐drugs (N05CD, N05CF)	3	2
Any analgesics (ATC M01A, M01B, N02A, N02B)	32	25
NSAID (ATC M01A, N02BA)	5	4
Opioids (ATC N02A)	6	5
Paracetamol (ATC N02BE01)	30	23
Any inhaler for respiratory conditions (ATC R03A, R03B)	15	12
Any PIMs defined by Beers Criteria with extensive list of anticholinergics^20^, ^22^	95	73
Any PIMs defined by 2024 Australian list^21^	45	35
Any PIMs defined by either list^20^, ^21^, ^22^	99	76
Psychotropic polypharmacy	16	12
Antidementia medication + psychotropic medication	21	16
Antidementia medication + PPI	15	12
Concurrent use of cholinesterase inhibitors and anticholinergics	28	22
NSAID (including low‐dose aspirin) + PPI	12	9

Abbreviations: NSAID, non‐steroid anti‐inflammatory drugs; PIMs, Potentially inappropriate medications; PPI, Proton pump inhibitor; SNRI, Serotonin–noradrenaline reuptake inhibitor; SSRI, Serotonin‐selective reuptake inhibitor; TCA, Tricyclic antidepressants.

^a^ Desvenlafaxine, Duloxetine, Venlafaxine.

^b^ Amitriptyline, Clomipramine, Dosulepin, Doxepin, Imipramine, Nortriptyline.

^c^ Agomelatine, Mianserin, Mirtazapine, Moclobemide, Reboxetine, Vortioxetine.

**TABLE 3:** Prescribing patterns of antidementia medications in different types of dementia.

	Alzheimer's disease (*n* = 44)	Frontotemporal dementia (*n* = 9)	Lewy body disease (*n* = 3)	Mixed (*n* = 6)	Vascular dementia (*n* = 17)	Others (*n* = 51)	Total (*n* = 130)
	*n* (%)	*n* (%)	*n* (%)	*n* (%)	*n* (%)	*n* (%)	*n* (%)
Any cholinesterase inhibitor (N06DA)	10 (23)	6 (67)	2 (67)	3 (50)	9 (53)	13 (26)	43 (33)
Donepezil (N06DA02)	8 (18)	6 (67)	1 (33)	1 (17)	6 (35)	11 (22)	33 (25)
Rivastigmine (N06DA03)	1 (2)	0 (0)	1 (33)	1 (17)	1 (6)	0 (0)	4 (3)
Galantamine (N06DA04)	1 (2)	0 (0)	0 (0)	1 (17)	2 (12)	2 (4)	6 (5)
Memantine (N06DX01)	2 (5)	0 (0)	1 (33)	0 (0)	2 (12)	1 (2)	6 (5)
Combination of Memantine and AChEi	1 (2)	0 (0)	1 (33)	0 (0)	1 (6)	1 (2)	4 (3)

Abbreviation: AChEi, Cholinesterase inhibitor.

### 3.3 Factors associated with medication use in people with dementia

The results of the logistic regression analyses for different medication exposures in people with dementia are reported in Table 4. People who were aged over 80 years had lower odds of using antidementia medications (adjusted odds ratio [aOR]: .29; 95% confidence interval [CI]: .12–.72). Polypharmacy was strongly associated with the use of psychotropics (aOR: 5.09; 95% CI: 1.94–13.39) and PIMs (aOR: 17.38; 95% CI: 5.12–59.02). A higher level of educational attainment (completed high school or above) was associated with lower odds of psychotropic usage (aOR: .33; 95% CI: .15–.76).

**TABLE 4:** Logistic regression model for medication exposure in participants living with dementia (*n* = 126)^a^.

	Any antidementia		Any psychotropics		Any PIMs in people with dementia	
	Odds ratio (95% CI)	*p* Value	Odds ratio (95% CI)	*p* Value	Odds ratio (95% CI)	*p* Value
Age >80 years old	**.29 (.12–.72)**	**.01**	.59 (.25–1.43)	.24	2.40 (.76–7.63)	.14
Male	1.86 (.76–4.55)	.18	.92 (.39–2.19)	.86	1.23 (.37–4.08)	.74
HSC or higher	.93 (.39–2.21)	.87	**.33 (.15–.76)**	**.01**	.30 (.09–1.02)	.05
Suggestive depression and depression	.67 (.25–1.81)	.42	1.90 (.74–4.89)	.18	2.03 (.55–7.54)	.29
Global deterioration scale: Moderate to moderately severe dementia (GDRS >4)	.70 (.25–2.01)	.51	1.33 (.49–3.62)	.58	1.21 (.29–5.09)	.79
Polypharmacy	2.05 (.76–5.47)	.15	**5.09 (1.94–13.39)**	**<.001**	**17.38 (5.12–59.02)**	**<.001**
Functional ability^b^	1.02 (.99–1.04)	.23	1.00 (.98–1.03)	.76	1.02 (.99–1.06)	.14
Better physical functioning^c^	1.39 (.51–3.73)	.52	1.68 (.64–4.40)	.29	.66 (.17–2.53)	.54
Better health‐related quality of life in participants with dementia^d^	9.84 (.76–127.74)	.08	.23 (.03–1.89)	.17	.25 (.01–6.48)	.40
Increased carer burden^e^	1.02 (.98–1.06)	.31	1.01 (.97–1.05)	.64	1.05 (.99–1.12)	.09
Better health‐related quality of life in carers^d^	5.74 (.58–57.00)	.14	.23 (.03–1.61)	.14	2.81 (.24–33.55)	.41

Abbreviations: DAD, Disability assessment for dementia; PIM, potentially inappropriate medication, defined as any use of 2019's Beers Medications or 2024's Australian list; SPPB, Short physical performance battery. Bold values indicate statistically significant findings.

^a^ Missing values were handled listwise.

^b^ Disability assessment for dementia was used as a measurement of functional ability in people with dementia and included in the logistic regression model as a continuous variable.

^c^ Physical functioning were characterised by SPPB in median split.

^d^ Health‐related quality of life characterised by EQ‐5D‐5L and included in the logistic regression model as a continuous variable.

^e^ Increased carer burden is characterised by Zarit burden index as a continuous variable.

## 4 DISCUSSION

To the best of our knowledge, this is the first community‐based study that examined the prevalence and potential factors associated with medication use in people with dementia in Australia. Antidementia, psychotropic and PIMs were used by 35%, 48% and 76% of people with dementia, respectively. Age, education and polypharmacy were associated with use of specific classes of medications in people with dementia.

The prevalence of antidementia medication use in people with dementia in this study was lower than in studies of community‐based people with dementia reported overseas, with 78% of people with dementia using antidementia medications in China.^11^ Similar to a previous Australian study using dispensing data from the Pharmaceutical Benefits Scheme (PBS), donepezil was the most commonly dispensed antidementia treatment.^10^ However, memantine was used by a larger proportion of participants using antidementia medications in our study (13% of antidementia users) compared with 8% found in the previous study.^10^ This disparity may be explained by the fact that the PBS only subsidises one medication treatment for dementia^10^; therefore, it may not capture the combination use of cholinesterase inhibitors and memantine, which was the case for the majority of memantine users (67% of memantine users) in this study. Furthermore, our study population was not representative of all people with dementia in Australia. Unsurprisingly, the prevalence of antidementia medication use in this population was higher than a previous Australian study of people with dementia living in RACFs.^9^ People living in RACFs tend to be more cognitively and functionally impaired, and may have different goals of care compared to those living in the community; and consideration of deprescribing of antidementia medications is recommended.^23^ While our study found that older age was associated with a reduced likelihood of using antidementia medications, results from previous studies have been mixed^12^; further research is required the investigate the relationship between age and the use of antidementia medications.

Psychotropics were one of the most commonly used medication classes in people with dementia. The prevalence of psychotropic usage in people with dementia included in this study was similar to previous studies.^11^, ^24^, ^25^ While the prevalence of antipsychotic use was similar to a recent study involving primary care patients with dementia in Australia,^26^ the use of antipsychotics was significantly lower in this population when compared to those living in Australian RACFs.^25^, ^27^ This may be because residents of RACFs have a higher prevalence of behavioural and psychological symptoms of dementia (BPSD), and antipsychotics are used as an addition to the management of BPSD when responses to non‐pharmacological approaches are inadequate.^2^ In the present study, people with higher educational level attainment had a lower likelihood of using psychotropics. Although recent studies have shown that lower education level was associated with increased use of antipsychotics in people with dementia,^28^ only a few studies included in a previous review identified education level as a predictor of psychotropic usage.^29^ The association between education level and psychotropic use may stem from socio‐economic status and health literacy. People with higher education levels tend to have better access to care and may have better health literacy, which may increase the knowledge and utilisation of other treatment, such as non‐pharmacological interventions.^30^ Further studies are required to examine the link between psychotropic utilisation in people with dementia and level of education.

Antihypertensives and lipid‐modifying agents were widely used in people with dementia even though consensus‐based guidelines suggest to consider ceasing certain preventative medications, such as lipid‐modifying agents and medications with longer potential time to benefit than patient's prognosis, to minimise the risk of polypharmacy.^31^ Polypharmacy was used by over half of the people with dementia included in this study and was associated with a 4 and 16 times higher odds of using psychotropics and PIMs, respectively. The association with psychotropics and PIMs may reflect the composition of polypharmacy, which is consistent with a previous study from Sweden.^32^ Our study was consistent with previous research showing polypharmacy was associated with exposure to PIMs.^4^ Furthermore, psychotropic polypharmacy was found to impact 12% of people with dementia, which is lower than the prevalence found in RACF residents with dementia in a meta‐analysis.^33^ This may be due to RACF residents being in a later stage of disease that increases the utilisation of psychotropics. In addition, non‐pharmacological intervention tends to be more time consuming, with a previous study indicating time as one of the greatest barriers in the utilisation of non‐pharmacological interventions,^34^ which resulted in the increased utilisation of pharmacological management of BPSD. Since polypharmacy and PIMs are associated with poor health outcomes,^5^ it is important to review and deprescribe these medications where appropriate.

### 4.1 Strengths and limitations

A broad range of medications, including over‐the‐counter medications, were collected by trial assessors through multiple sources such as interviews with people with dementia and carers, and observation of medication packages and lists; therefore, we are confident of the accuracy of the medication data collected. While previous studies investigated the prevalence and factors associated with medication use in people with dementia, the present study also investigated carer characteristics, which are often understudied. Furthermore, the present study includes private prescriptions that were not subsidised by PBS, which provides a more comprehensive understanding of medications used by people with dementia compared to studies using PBS data as a primary data source.

This study has a number of limitations. As this was a cross‐sectional study, we were limited in our ability to make causal inferences about exposures and outcomes of interest. Also, this study had a small sample size, which likely impacted the accuracy of the effect sizes observed, evidenced by some wide confidence intervals for some measures. Moreover, as this study used data from a randomised‐controlled trial, there were strict inclusion criteria for participants with dementia. Participants were generally in mild or moderate stages of dementia, and therefore, are unlikely to be generalisable to people with more severe dementia. Also, participants of this study were recruited from Sydney, Australia; therefore, this may limit generalisability to other places in Australia and internationally.

## 5 CONCLUSIONS

Quality use of medicines is important for improving quality of life and well‐being in people with dementia. Our study demonstrates high use of polypharmacy and PIMs in people with dementia in the community, with age, polypharmacy and education status being factors associated with the use of certain medication classes. Future research should focus on identifying and developing appropriate strategies for reducing the burden of inappropriate polypharmacy and investigating the potential influence of health literacy on psychotropic use in people with dementia. Furthermore, additional research is required into the prescribing trends and outcomes of psychotropic use in people with different types and stages of dementia in Australia. Clinicians should regularly review medication use in people with dementia to ensure the quality and judicious use of medications.

## FUNDING INFORMATION

I‐HARP is supported by NHMRC under the Boosting Dementia Research Grants Scheme (APP1137050: 2016 Priority 1 Implementation of Dementia Research into Clinical Practice and Care). ECKT is supported by a Dementia Australia Research Foundation Mid‐Career Research Fellowship.

## CONFLICT OF INTEREST STATEMENT

No conflicts of interest declared.

## Supporting information


Tables S1–S3

## Data Availability

Research data are not shared.
