# Supplementary material for: Prescribing patterns in people living with dementia in the community: A cross‐sectional study
Source: Australas J Ageing. 2024 Oct 27;44(1):e13380. doi: 10.1111/ajag.13380 (PMC11752786; doi:10.1111/ajag.13380)
Supplement: Supplementary file 1 — Tables S1–S3 [file AJAG-44-0-s001.docx]

**Supplementary Table 1:** ATC code for medication included

|  | ATC code |
| --- | --- |
| Antidementia | N06D |
| Psychotropics  Antidepressants  Antipsychotics  Antiepileptic  Anxiolytics  Hypnotics and sedatives  Opioids | N06A  N05A  N03A  N05B  N05C  N02A |
| Cardiovascular medications | C |
| Analgesics | M01A  M01B  N02A  N02B |

**Supplementary Table 2:** Prevalence of medication use by ATC level 1 (n=130)

| ATC level 1 | Description | n | % |
| --- | --- | --- | --- |
| ATC C | Any cardiovascular system | 103 | 79 |
| ATC N | Any nervous system | 94 | 72 |
| ATC A | Any alimentary tract and metabolism | 82 | 63 |
| ATC B | Any blood and blood forming organs | 65 | 50 |
| ATC M | Any musculoskeletal system | 21 | 16 |
| ATC R | Any respiratory system | 20 | 15 |
| ATC G | Any genitourinary system and sex hormones | 18 | 14 |
| ATC H | Any systemic hormonal preparations, excluding sex hormones and insulins | 14 | 11 |
| ATC S | Any sensory organs | 11 | 9 |
| ATC L | Any antineoplastic and immunomodulating agents | 5 | 4 |
| ATC J | Any anti-infectives for systemic use | 5 | 4 |
| ATC D | Any dermatologicals | 4 | 3 |
| ATC P | Any antiparasitic products, insecticides and repellents | 3 | 2 |
| ATC V | Any various | 0 | 0 |

**Supplementary Table 3:** Top 20 most frequently used medications by people with dementia included^a^

| Active ingredients | ATC | n | % |
| --- | --- | --- | --- |
| B01AC06 | Aspirin | 37 | 29 |
| C10AA07 | Rosuvastatin | 35 | 27 |
| N06DA02 | Donepezil | 33 | 25 |
| N02BE01 | Paracetamol | 30 | 23 |
| C10AA05 | Atorvastatin | 24 | 19 |
| A02BC02 | Pantoprazole | 16 | 12 |
| C09CA04 | Irbesartan | 15 | 12 |
| B01AC04 | Clopidogrel | 14 | 11 |
| A10BA02 | Metformin | 13 | 10 |
| C03CA01 | Furosemide | 13 | 10 |
| A02BC05 | Esomeprazole | 12 | 9 |
| C07AB02 | Metoprolol | 12 | 9 |
| N06AX11 | Mirtazapine | 12 | 9 |
| C08CA01 | Amlodipine | 10 | 8 |
| H03AA01 | Levothyroxine | 10 | 8 |
| N06AB10 | Escitalopram | 9 | 7 |
| C09AA04 | Perindopril | 8 | 6 |
| C09CA07 | Telmisartan | 8 | 6 |
| C10AA01 | Simvastatin | 8 | 6 |
| A10BB09 | Gliclazide | 7 | 5 |
| C01AA05 | Digoxin | 7 | 5 |
| C01DA14 | Isosorbide Mononitrate | 7 | 5 |
| M04AA01 | Allopurinol | 7 | 5 |

^a^More than 20 medications were listed as gliclazide; digoxin, isosorbide mononitrate and allopurinol have the same frequency.
